# Luteoloside Induces G0/G1 Phase Arrest of Neuroblastoma Cells by Targeting p38 MAPK

**DOI:** 10.3390/molecules28041748

**Published:** 2023-02-12

**Authors:** Ya He, Maohong Luo, Shan Lei, Zhirui Zeng, Tengxiang Chen, Yingmin Wu, Dongyan Wang, Long Wang, Lu Wang

**Affiliations:** Guizhou Provincial Key Laboratory of Pathogenesis and Drug Research on Common Chronic Disease, Department of Physiology, College of Basic Medicine, Guizhou Medical University, Guiyang 550025, China

**Keywords:** neuroblastoma, luteoloside, p38 MAPK, proliferation, cell cycle arrest

## Abstract

Luteoloside has shown anti-inflammatory, antiviral, and antitumor properties. However, the effect and mechanism of luteoloside on neuroblastoma cells remain unknown. The proliferation of human neuroblastoma cells (SH-SY5Y and SK-N-AS) treated with different concentrations of luteoloside (0, 12.5, 25, and 50 μM) was detected by the MTT assay and colony formation assay. Cell apoptosis and cell cycle were examined by Hoechst staining and flow cytometry. A subcutaneous tumorigenesis model was established in nude mice to evaluate the effect of luteoloside on tumor growth in vivo. Bioinformatics, molecular docking techniques, and cellular thermal shift assays were utilized to predict the potential targets of luteoloside in neuroblastoma. The p38 MAPK inhibitor SB203580 was used to confirm the role of p38 MAPK. Luteoloside inhibited the proliferation of neuroblastoma cells in vitro and in vivo. Luteoloside slightly induced cellular G0/G1 phase arrest and reduced the expression levels of G0/G1 phase–related genes and the proteins cyclin D1, CDK4, and C-myc, which are downregulated by p38 MAPK pathways. Meanwhile, p38 was identified as the target of luteoloside, and inhibition of p38 MAPK reversed the inhibitory effect of luteoloside on neuroblastoma cells. Luteoloside is a potential anticancer drug for treating neuroblastoma by activating p38 MAPK.

## 1. Introduction

Neuroblastoma is one of the most malignant tumors in peripheral neuroma and often occurs in children under 5 years old, which makes neuroblastoma hard to diagnose and treat [1,2]. In spite of considerable progress in molecular mechanisms and therapeutic approach, survival rates remain low. Moreover, because of the high toxicity of radio-chemotherapy and the age of the patients, it is difficult to achieve therapeutic success [3,4]. Furthermore, oncology drugs have the lowest likelihood of approval in phase I (6.7%) compared with drugs treating other pediatric diseases [5]. Consequently, there is an urgent need to discover novel therapeutic agents that are well tolerated and have low toxicity to treat neuroblastoma. In recent years, researchers have found that traditional Chinese medicines, including flavonoids, exhibit significant antitumor effects and have begun clinical trials [6,7].

Luteoloside, a natural flavonoid substance also known as luteolin 7-O-glucoside, exhibits a variety of biological activities, such as anti-inflammatory [8], antiviral [9], cardiovascular protection [10], and anti-tumor activity [11,12,13,14]. Researchers found that luteoloside suppressed oral cancer cell migration by regulating matrix metalloproteinase-2 expression, and inhibited human nasopharyngeal carcinoma cell proliferation and promoted apoptosis [11,12]. Other researchers reported that luteoloside inhibited proliferation and modulated apoptosis by mitogen-activated protein kinase (MAPK) and mammalian target of rapamycin (mTOR) signaling pathways in human cervical cancer [13]. Our previous research suggests that luteoloside promoted the apoptosis in breast cancer cells [14]. In addition, some reports showed that luteolin, which has a similar structure to luteoloside, enhanced neuronal antioxidant defense capacity and suppressed glioma cell proliferation by inducing apoptosis and autophagy via MAPK activation [15,16].

The structure of a drug usually determines its function. This suggests that the molecular structure of luteoloside can be used to infer its mechanism of action. Network pharmacology and molecular docking are effective methods in drug design and screening, and are being used to explore the targets and mechanisms of traditional Chinese medicine [17,18]. Network pharmacology is a new discipline based on the theory of systems biology, which can predict the targets of drug action based on the target characteristics of drugs and diseases and their network relationships [19]. It is commonly used in research on multitarget and multiaction traditional Chinese medicine. Molecular docking allows a receptor–ligand complex to be docked according to an analysis of the specific receptor docking sites and the drug molecule [20]. Although studies have shown that luteoloside has anti-tumor effects, the specific targets are still unclear, and neuroblastoma has not yet been studied to determine the effects and mechanisms of luteoloside. Therefore, we may predict the possible molecules of action of luteoloside using network pharmacology and molecular docking techniques.

In this study, we attempted to elucidate the specific biological activity and crucial molecular mechanisms of luteoloside on neuroblastoma, possibly assisting in the treatment of neuroblastoma. We show that luteoloside targets to p38 MAPK and induces cell cycle arrest, which leads to the inhibition of neuroblastoma cell proliferation. This study shows that luteoloside exerts anti-neuroblastoma activity in a novel manner, and offers a promising approach for neuroblastoma treatment.

## 2. Results

### 2.1. Luteoloside Inhibits Proliferation of Neuroblastoma Cell

**Figure 1:**
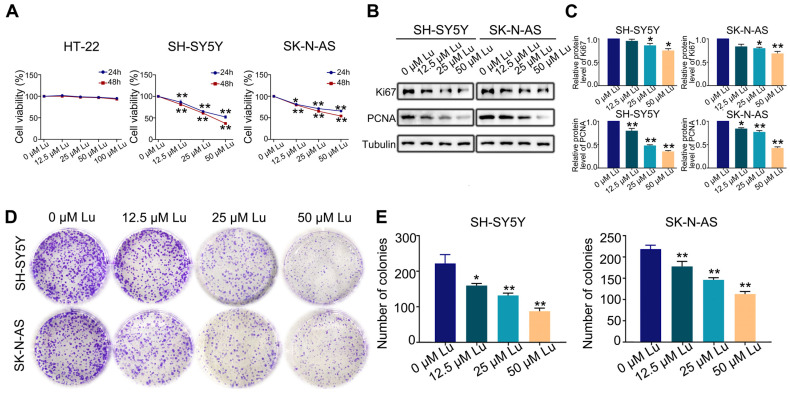
Luteoloside inhibits the proliferation of neuroblastoma cells. SH-SY5Y and SK-N-AS cells were treated with varying concentrations (0, 12.5, 25, and 50 μM) of luteoloside for 24 or 48 h, while HT-22 was treated with different concentrations (0, 12.5, 25, 50, and 100 μM) of luteoloside for the same periods. (**A**) The viability of HT-22, SH-SY5Y, and SK-N-AS cells was detected in each group. (**B**) The expression of PCNA and Ki67 was tested by Western blot. (**C**) Statistical analysis of the expressions intensity of PCNA, Ki67 in SH-SY5Y and SK-N-AS cells. Tubulin is taken as internal reference. (**D**) The colony formation of SH-SY5Y and SK-N-AS cells was tested in each group. (**E**) Statistical analysis of the number of colonies in each group. *, *p* < 0.05; **, *p* < 0.01. *n* = 3. The control group is used for comparison. Data are shown as mean ± SD.

The first set of questions aimed to evaluate the proliferative effect of luteoloside on HT-22, SH-SY5Y, and SK-N-AS cells. SH-SY5Y and SK-N-AS cells were treated with varying concentrations (0, 12.5, 25, and 50 μM) of luteoloside for 24 and 48 h, while HT-22 cells were treated with different concentrations (0, 12.5, 25, 50, and 100 μM), also for 24 and 48 h. MTT assays revealed that luteoloside obviously decreased the proliferation rate of SH-SY5Y and SK-N-AS cells in a dose-dependent manner, but had no significant effect on normal cells of HT-22 (Figure 1A). The protein expressions of the cell proliferation markers Ki-67 and PCNA were both downregulated by luteoloside (Figure 1B,C). Additionally, colony formation assays demonstrated that SH-SY5Y and SK-N-AS exhibited low colony formation capacity after being treated with luteoloside (Figure 1D,E). 

### 2.2. Luteoloside Slightly Improves Neuroblastoma Cell Apoptosis

**Figure 2:**
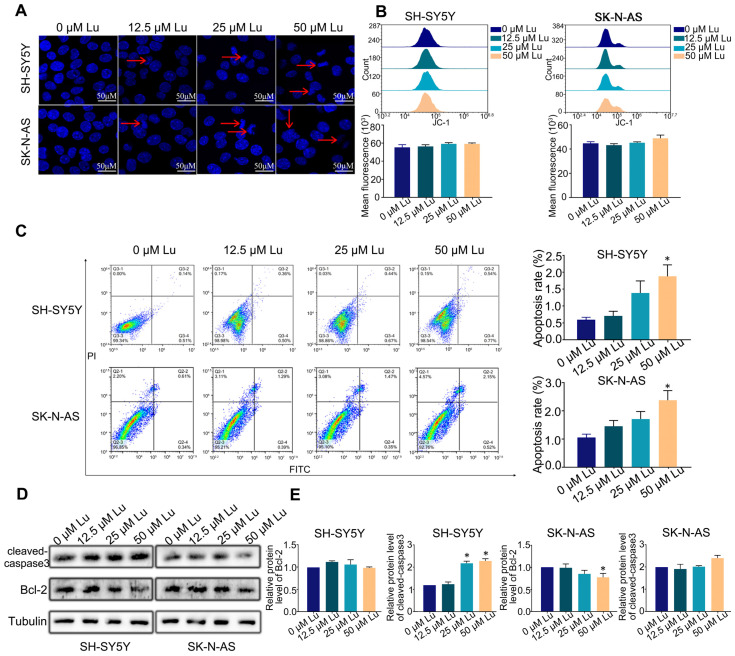
Effect of luteoloside on the neuroblastoma cell apoptosis. SH-SY5Y and SK-N-AS cells were treated with varying concentrations (0, 12.5, 25, and 50 μM) of luteoloside for 24 h. (**A**) Cell morphology with Hoechst 33258 staining was observed under a fluorescence microscope. Red arrows show the apoptotic bodies. Bar = 50 μM. (**B**) Flow cytometry was used to detect the change of mitochondrial membrane potential (MMP) in each group. (**C**) Apoptosis distribution of SH-SY5Y and SK-N-AS cells was tested by flow cytometry. (**D**) The protein expressions of cleaved-caspase3 and Bcl-2 were tested by Western blot. (**E**) Statistical analysis of the expression intensity of cleaved caspase-3 and Bcl-2 in SH-SY5Y and SK-N-AS cells. Tubulin is taken as internal reference. *, *p* < 0.05. *n* = 3. The control group is used for comparison. Data are shown as mean ± SD.

To assess the effect of luteoloside on the apoptosis of human neuroblastoma cells, cells were stained with Hoechst 33258 after being treated with different concentrations (0, 12.5, 25, and 50 μM) of luteoloside for 24 h. The cell nuclei of each group were uniformly stained, and the edge contours were clear; only a few cell nuclei of the 25 and 50 μM groups appeared with apoptotic bodies (Figure 2A). There were no significant changes of mitochondrial membrane potential (MMP) in either SH-SY5Y and SK-N-AS cells (Figure 2B). The apoptosis rates of SH-SY5Y and SK-N-AS cells were not significantly changed at in the 0, 12.5, or 25 μM luteoloside-treated groups, but were increased in the 50 μM luteoloside-treated groups (Figure 2C). Western blot results demonstrated that the expression levels of the Bcl-2 protein had no change in SH-SY5Y cells in each luteoloside-treated groups, and the levels of cleaved-caspase3 in the 25 and 50 μM groups were increased. As for SK-N-AS cells, the expression levels of Bcl-2 in the 25 and 50 μM luteoloside-treated groups were less than those of the control group (Figure 2D,E). These results indicate that luteoloside slightly induced apoptosis of neuroblastoma cells, but the inhibitory effect of luteoloside on human neuroblastoma cell proliferation is not primarily mediated by apoptosis.

### 2.3. Luteoloside Induces Neuroblastoma Cell Cycle Arrest

**Figure 3:**
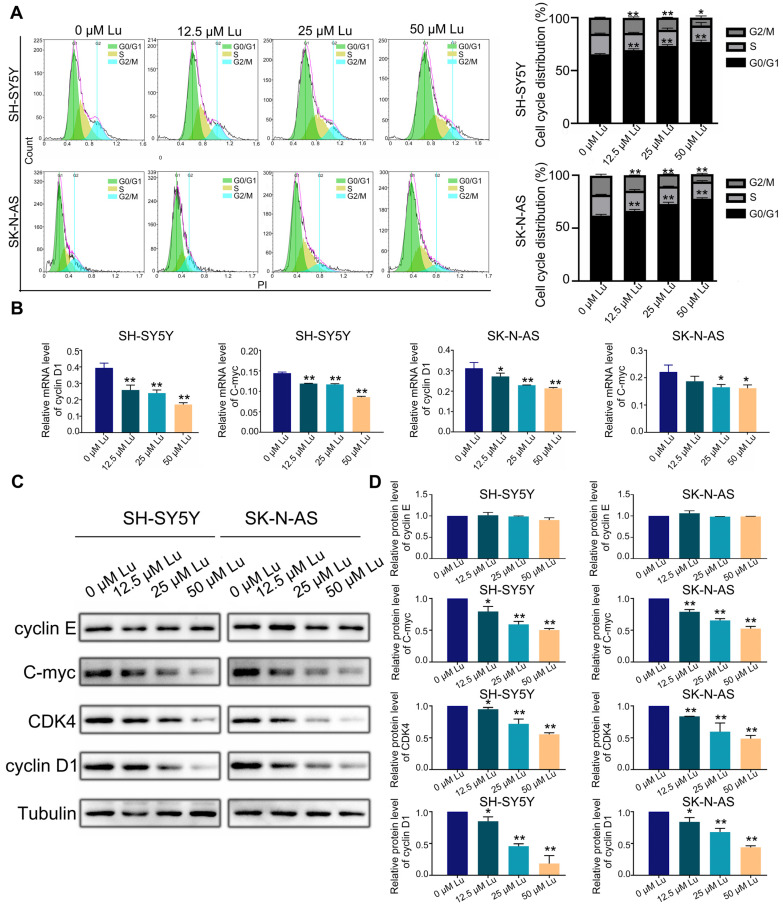
Luteoloside induces neuroblastoma cell cycle arrest. SH-SY5Y and SK-N-AS cells were treated with varying concentrations (0, 12.5, 25, and 50 μM) of luteoloside for 24 h. (**A**) The SH-SY5Y and SK-N-AS cell cycle distributions were measured by flow cytometric analysis. (**B**) The protein expressions of cyclin D1, cyclin E, C-myc, and CDK4 were tested by Western blot. (**C**) Statistical analysis of the expression intensity of cyclin D1, cyclin E, C-myc, and CDK4 in SH-SY5Y and SK-N-AS cells. Tubulin is taken as internal reference. (**D**) The relative mRNA expression of cyclin D1 and C-myc in SH-SY5Y and SK-N-AS cells was analyzed using qRT-PCR. *, *p* < 0.05; **, *p* < 0.01. *n* = 3. The control group is used for comparison. Data are shown as mean ± SD.

Further, we analyzed the cell cycle and related protein expression to explore the impact of luteoloside on DNA replication and cell cycle processes in neuroblastoma cells. The flow cytometry results revealed that luteoloside elevated the percentage of neuroblastoma cells in the G0/G1 phase, but significantly reduced the cell numbers in the G2 phase (Figure 3A). Western blot assay results demonstrated that the expression levels of the G1 phase–related proteins cyclin D1 and cyclin-dependent kinase 4 (CDK4) decreased after treatment with luteoloside. However, the expression of cyclin E proteins associated with the late G1 phase was not significantly changed. The expression level of C-myc was reduced in the luteoloside-treated group (Figure 3B,C). Meanwhile, the mRNA levels of cyclin D1 and C-myc were slightly decreased compared with the control groups (Figure 3D). These results show that luteoloside induces G0/G1 phase arrest in neuroblastoma cells by regulating the proteins cyclin D1 and C-myc. 

### 2.4. Luteoloside Suppressed the Tumor Growth In Vivo

**Figure 4:**
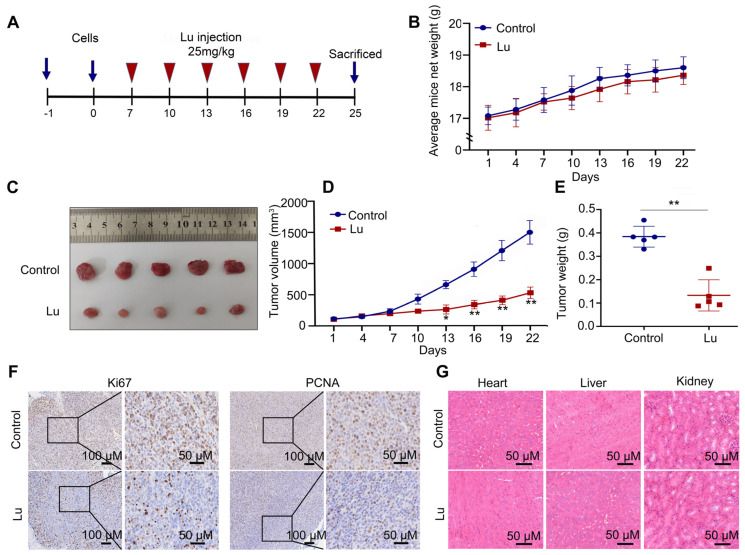
Luteoloside suppressed tumor growth in vivo. (**A**) The process sketch diagram. (**B**) The average mouse weight treated with DMSO or luteoloside. (**C**–**E**) Tumor volume and weight of tissues treated with DMSO or luteoloside. (**F**) The expression of Ki67 and PCNA in neuroblastoma and adjacent tissues was analyzed using immunohistochemical staining (IHC). (**G**) Hematoxylin–eosin (HE) was used to evaluate toxicity to the heart, liver, and kidney after treatment with luteoloside. *, *p* < 0.05; **, *p* < 0.01. *n* = 5. The control group is used for comparison. Data are shown as mean ± SD.

We also used female BALB/c nude mice as a subcutaneous tumorigenesis model to evaluate the antitumor potential of luteoloside in vivo (Figure 4A). Tumor volume and weight in the luteoloside-treated group were obviously decreased compared with the control group (Figure 4B–E). Immunohistochemical staining (IHC) results showed that neuroblastoma tissues treated with luteoloside had low expression of PCNA and Ki67 (Figure 4F). Luteoloside did not damage the heart, liver, or kidney (Figure 4G). These results suggest that luteoloside had the same inhibitory effect and no toxicity in vivo.

### 2.5. Luteoloside Induces Neuroblastoma Cell Cycle Arrest through the p38 MAPK Signaling Pathway

**Figure 5:**
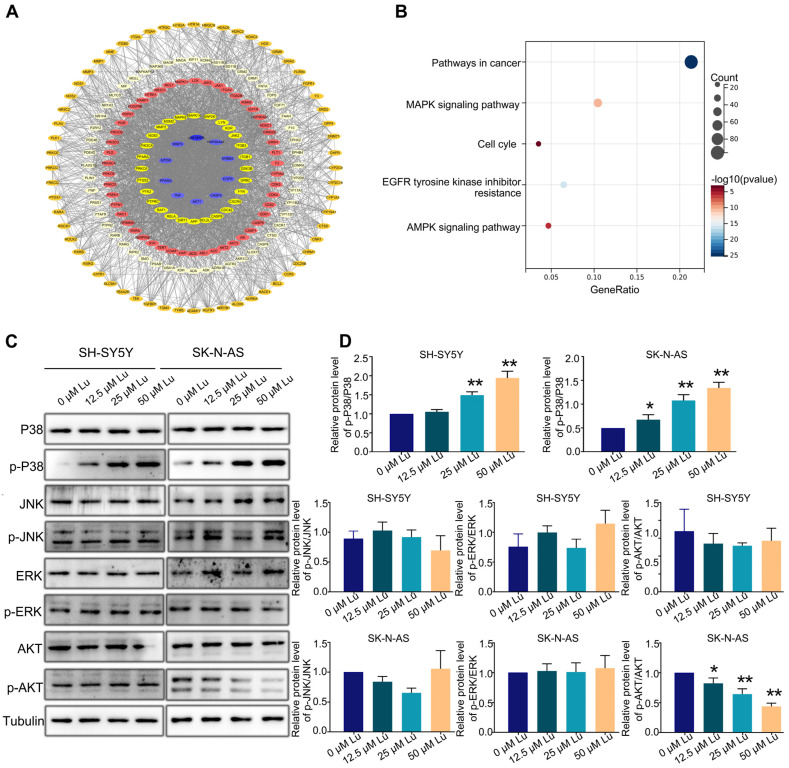
Luteoloside induces neuroblastoma cell cycle arrest through the p38 MAPK signaling pathway. SH-SY5Y and SK-N-AS cells were treated with different concentrations (0, 12.5, 25, and 50 μM) of luteoloside for 24 h. (**A**) The protein–protein interaction (PPI) network of potential luteoloside targets. (**B**) Kyoto Encyclopedia of Genes and Genomes (KEGG) pathway analysis. (**C**) The protein expressions of JNK, p-JNK, ERK, p-ERK, p38, p-P38, AKT, and p-AKT were tested by Western blot. (**D**) Statistical analysis of the expression intensity of p-AKT/AKT, p-JNK/JNK, p-ERK/ERK, p-P38/P38 in SH-SY5Y and SK-N-AS cells. *, *p* < 0.05; **, *p* < 0.01. *n* = 3. The control group is used for comparison. Data are shown as mean ± SD.

Next, we predicted the potential targets of luteoloside by using SwissTargetPrediction (http://swisstargetprediction.ch/) to identify the possible pathways involved in luteoloside-induced neuroblastoma cell cycle arrest; 495 potential targets were found (Figure 5A). Furthermore, using Kyoto Encyclopedia of Genes and Genomes (KEGG, https://david.ncifcrf.gov/tools.jsp) pathway analysis, we found that most of those targets were significantly enriched in cancer, MAPK signaling pathways and cell cycles (Figure 5B). We analyzed the expression levels of MAPK and Akt (protein kinase B) signaling pathway proteins, including AKT, p-AKT, c-Jun N-terminal kinase (JNK), p-JNK, extracellular regulated protein kinases (ERK), p-ERK, p38, and p-p38. The expression level of p-AKT was downregulated in SK-N-AS cells (Figure 5C). The protein level of p-p38 in was significantly upregulated in the luteoloside-treated groups compared with the control group (Figure 5C). Given these results, the activation of the p38 MAPK signaling pathway plays an essential role in the effect of luteoloside on neuroblastoma.

### 2.6. P38 MAPK May Be a Key Target of Luteoloside

**Figure 6:**
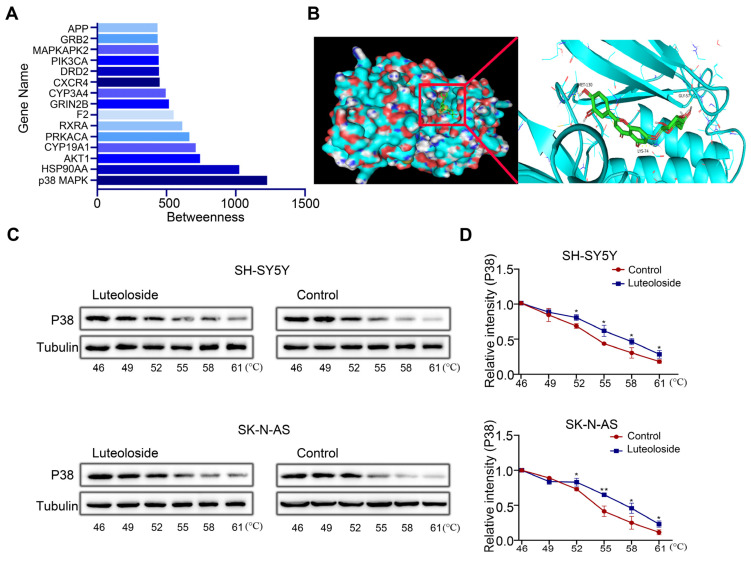
P38 MAPK was identified as a key target of luteoloside. (**A**) The top 15 luteoloside targets were visualized using Cytoscape. (**B**) The binding mode of luteoloside with p38 MAPK and the 3D illustration of the details of the interaction. Green shows luteoloside, aquamarine shows the protein of p38 MAPK. (**C**) The cellular thermal shift assay was used to evaluate luteoloside–p38 MAPK interactions in SH-SY5Y and SK-N-AS cells. (**D**) Quantification of the relative intensity of the p38 MAPK protein versus increased temperature. Tubulin is taken as internal reference. *, *p* < 0.05; **, *p* < 0.01. *n* = 3. The control group is used for comparison. Data are shown as mean ± SD.

Finding a drug’s target is key to drug research. To further explore the target of luteoloside, we evaluated the betweenness centrality of these targets, and found that p38 MAPK had the highest betweenness centrality (Figure 6A). Then, we analyzed the binding model of luteoloside and the p38 MAPK protein by using molecular docking technology. The 3D drawing showed that luteoloside can bind to the residues Gly57, Lys74, and Met-130 of the p38 MAPK protein, exhibiting a stabilizing binding conformation (Figure 6B). The correlation between luteoloside and p38 MAPK was tested using a CETSA experiment, and it was found that after treatment with luteoloside, the thermal stability of p38 MAPK was increased compared with the control group (Figure 6C,D). These results suggest that p38 MAPK may be a key target of luteoloside. 

### 2.7. P38 MAPK Inhibitor Reversed Proliferate Inhibition and Cell Cycle Arrest Induced by Luteoloside in Neuroblastoma Cells 

**Figure 7:**
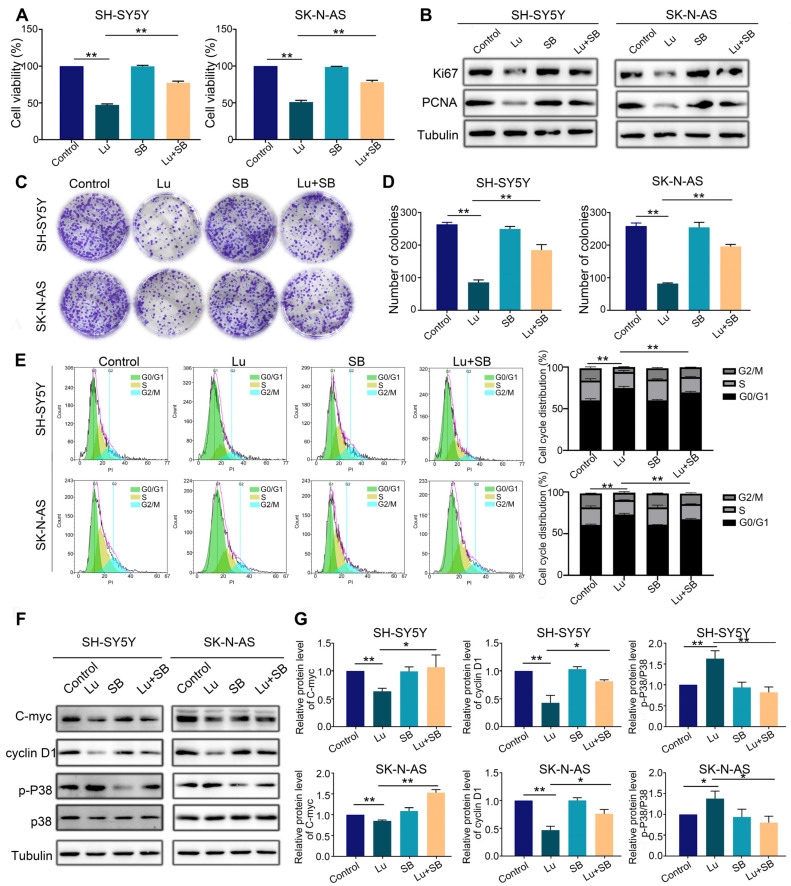
Inhibition of the p38 MAPK signaling pathway reversed cell cycle arrest caused by luteoloside on neuroblastoma cells. SH-SY5Y and SK-N-AS cells were treated with 0 μM luteoloside (control group), 50 μM luteoloside (Lu group), 20 μM SB203580 (SB group), and luteoloside + SB203580 (Lu + SB group) for 24 h. (**A**) The viability of SH-SY5Y and SK-N-AS cells was detected in each group. (**B**) The protein expressions of PCNA and Ki67 were tested by Western blot. (**C**) The colony formation of SH-SY5Y and SK-N-AS cells was tested in each group. (**D**) Statistical analysis of the number of colonies in each group. (**E**) SH-SY5Y and SK-N-AS cell cycle distributions were measured by flow cytometry in each group. (**F**) The expression of cyclin D1, C-myc, p38, and p-P38 was tested by Western blot. (**G**) Statistical analysis of the expression intensity of cyclin D1, C-myc, and p-P38/p38 in SH-SY5Y and SK-N-AS cells. Tubulin is taken as internal reference. *, *p* < 0.05; **, *p* < 0.01. *n* = 3. The control group is used for comparison. Data are shown as mean ± SD.

Finally, to confirm that p38 MAPK is the target of luteoloside, we utilized a specific p38 MAPK inhibitor, SB203580 (20 μM), to pretreat SH-SY5Y and SK-N-AS cells for 2 h, followed by combined treatment with luteoloside (50 μM) for 24 h. MTT assays revealed that the SB203580 pretreated group could partially recover from proliferation inhibition compared with the group treated with luteoloside alone (Figure 7A). The protein expression of the cell proliferation markers Ki-67 and PCNA was upregulated by luteoloside in the SB203580 pretreated group (Figure 7B). Additionally, colony formation assays demonstrated that SH-SY5Y and SK-N-AS partially recovered colony formation capacity when pretreated with SB203580 (Figure 7C,D). The results showed that SB203580 pretreatment could partially reverse G1 phase arrest from luteoloside (Figure 7E). Additionally, the p-P38 level of the SB203580 pretreated groups was clearly downregulated, while the levels of cyclin D1 and C-myc were clearly upregulated (Figure 7F,G). These data further reveal that luteoloside induces neuroblastoma cell cycle arrest via the p38 MAPK signaling pathway.

## 3. Discussion

Traditional therapeutic strategies for neuroblastoma that utilize radio-chemotherapy, bone marrow transplantation, and immunotherapy still result in many side effects [21,22]. Safer and better-tolerated therapies and new drugs are urgently needed. This study found that luteoloside inhibited the proliferation of neuroblastoma cells through G0/G1 phase arrest. We revealed that luteoloside targets p38 MAPK by increasing the phosphorylation level, thereby downregulating the expression of key downstream proteins of the G1 phase, such as C-myc, cyclin D1, and CDK4, eventually causing G0/G1 phase arrest.

A disruption of cell cycle progression is one of the basic mechanisms of tumorigenesis. Cells increase in number through repeated cycles of division, which is called the cell cycle. The cycle includes two parts: the interphase consisting of the G1, S, and G2 phases and the mitosis phase. Disturbances of the cell cycle and its regulatory systems commonly lead to uncontrolled cell proliferation and, eventually, carcinogenesis [23,24]. Therefore, controlling the cell cycle is key to suppressing tumor cell proliferation. Cyclins and cyclin-dependent kinases (CDKs) are responsible for the progression of cells past various checkpoints. Cyclin D1 is a regulatory subunit of CDK4 or CDK6 that binds with CDK4/6 to form a complex. This complex triggers the activation of genes related to DNA replication to ensure that cells transition from the G1 phase checkpoint to the S phase [25]. Some studies have indicated that the overexpression of cyclin D1 or CDK4 caused tumor in mice [26,27,28], while knockout cyclin D1 or CDK4 caused resistance to the development of murine breast cancer [29]. Deletion of CDK4 caused cell cycle arrest and promoted cell senescence in lung cancer cells with the endogenous K-RAS oncogene [30]. Several clinical studies on inhibitors targeting CDK4 and CDK6 in pRb-positive tumors (breast cancer, melanoma, liposarcoma, and so on) have shown that the cyclin D–CDK4/6–pRb axis has a significant impact on tumor proliferation and transformation [31]. These studies suggest that regulators of the expressions of cell cycle proteins could be developed for neuroblastoma treatment. Many researchers have reported that natural products, including artemisinin, olive leaves extract, and oleuropein, can inhibit the proliferation of neuroblastoma cells by mediating cell cycle [32,33,34,35]. Similar mechanisms for luteoloside were also found by Zhou et al. They found that luteoloside caused G0/G1 arrest and autophagy through the AKT/mTOR signaling pathway in human non–small cell lung cancer, but this process does not effectively lead to apoptotic cell death [36]. In this study, we observed a small amount of apoptosis and increased the expression of cleaved-caspase3 induced by luteoloside. This may be due to luteoloside regulation of other pathways or proteins, which requires further study. In addition, we revealed that SH-SY5Y and SK-N-AS cells were arrested in the G0/G1 phase, and the expression of the cyclin D1 and CDK4 proteins was significantly decreased after treatment with luteoloside. 

In recent years, the emerging field of network pharmacology has had a significant impact on traditional Chinese medicine [37]. An important part of network pharmacology is the forecasting of the molecular mechanisms of drugs based on complex biological network models and interactions between targets and diseases [38,39]. We predicted the potential targets of luteoloside using SwissTargetPrediction, and KEGG pathway analysis showed that the 495 targets were significantly enriched in the MAPK signaling pathway. The MAPK signaling pathway is an essential pathway in cell growth. It includes the ERK, p38 MAPK, and JNK subfamilies [40]. ERK exists widely in tissues and participates in the regulation of cell proliferation and differentiation; JNK is a vital signal molecule in cellular signal transduction; and p38 MAPK consists of five members (p38α, p38β1, p38β2, p38γ, and p38δ) that mediate apoptosis and inflammation [41], and can either inhibit tumor cell homeostasis, proliferation, differentiation, and apoptosis, or enhance tumor development and metastasis. Our results show that luteoloside could bind to the residues Gly57, Lys74, and Met 130 of p38 MAPK, and this interaction was confirmed by the CETSA experiment. Furthermore, an upregulating p-p38 MAPK was observed in the luteoloside-treated group. We speculate that this may be due to the binding of luteoloside and p38 MAPK, inducing increased activity of p38, but the specific structural mechanism needs further investigation. Some studies have clarified the role of the p38 MAPK signaling pathway in various tumors, providing theoretical guidance for p38 MAPK as a potential target of cancer therapy [42,43,44]. A report demonstrated that the flavonoid isoliquiritigenin exhibited antiproliferative effects in neuroblastoma cells by upregulating p-p38 MAPK [45]. Other research showed that notoginsenoside induced G2/M phase arrest and apoptosis by the activation of p38 MAPK [46]. 

The activation of p38 MAPK regulates cell cycle progression at both the G1/S and G2/M transitions through multiple mechanisms, including the downregulation of cyclins [47]. Cyclin D1, as the main regulator of the G1 phase of the cell cycle, promotes progression to the S phase and is downregulated by the activation of p38 MAPK [48]. In our study, the gene and protein expression levels of cyclin D1 were downregulated by luteoloside, and p38 MAPK phosphorylation was remarkably increased. This effect was reversed after by treatment with p38 MAPK inhibitor SB203580. Interestingly, we found that C-myc was decreased by luteoloside in neuroblastoma, which was reversed by the inhibition of p38 MAPK. C-myc is an important transcription factor involved in cell cycle control and tumorigenesis that can promote G1–S progression of the cell cycle and is regulated by p38 MAPK [49]. C-myc has been shown to collaborate with cyclin D1, N-myc, or L-myc to induce lymphomas in transgenic mice [50]. Although it is clear that cyclin D1 and C-myc were downregulated by luteoloside in this study, the relationship between these two needs to be further investigated.

In this study, p38 MAPK was identified as a potential target of luteoloside, and p38 MAPK phosphorylation was remarkably increased by luteoloside. Furthermore, cell cycle arrest caused by luteoloside in neuroblastoma cells could be reversed by the inhibition of p38 MAPK. These data reveal that luteoloside induces neuroblastoma cell cycle arrest via the p38 MAPK signaling pathway. In summary, our study shows that luteoloside has anti- neuroblastoma activity and potential applications against human neuroblastoma cells.

## 4. Materials and Methods

### 4.1. Cell Culture

SH-SY5Y and HT-22 cell lines were purchased from DSMZ (Braunschweig, Germany); SK-N-AS cell line was purchased from Procell Life Science&Technology Co. (Wuhan, China). SH-SY5Y cells were cultured in DMEM/F12 (1:1), while HT-22 and SK-N-AS cells were cultured in DMEM (Gibco, Grand Island, NY, USA) with 10% FBS (BI, Kibbutz Beit Haemek, Israel) and 1% penicillin/streptomycin (Invitrogen; Carlsbad, CA, USA) at 37 °C with 5% CO_2_. Luteoloside (HY-N0540) and SB203580 (HY-10256) were purchased from MedChemExpress (MCE, Wuhan, China). Cells were treated with different concentrations of luteoloside (0, 12.5, 25, and 50 μM) for 24 h unless otherwise specified.

### 4.2. MTT Assay

SH-SY5Y, SK-N-AS, and HT-22 cells were cultured in 96-well plates with a density of 1 × 10^4^/well. Different concentrations (0, 12.5, 25, and 50 μM) of luteoloside were used to treat the SH-SY5Y and SK-N-AS cells for 24 or 48 h, while HT-22 was treated with 0, 12.5, 25, 50, and 100 μM of luteoloside. An amount of 20 µL of MTT solution (M1020, Solarbio, Beijing, China) was added to incubate cells in each well for 4 h; then DMSO (D8370, Solarbio, Beijing, China) was used to stop the reaction. Cell proliferation rates were measured using a spectrophotometer (BioTek, Winooski, VT, USA) set at 490 nm.

### 4.3. Colony Formation Assay

SH-SY5Y and SK-N-AS cells were cultured in 6-well plates (1000 cells/well). Different concentrations (0, 12.5, 25, and 50 μM) of luteoloside were used to treat the cells. After 10 days, a 4% paraformaldehyde solution (BL539A, Biosharp, Hefei, China) was used to immobilize the cells, and a 0.5% crystal violet solution (G1064, Solarbio, Beijing, China) was used to stain. Three replicates were used for each treatment group.

### 4.4. Flow Cytometry Analysis

SH-SY5Y cells and SK-N-AS cells were cultured with different concentrations (0, 12.5, 25, and 50 μM) of luteoloside in 6-well plates. Cells were subjected to serum starvation for about 24 h for cell cycle synchronization. Subsequently, cells were resuspended in DMEM with 10% FBS and fixed in 70% ethanol overnight at −20 °C. After washing the cells three times with PBS and collecting them, the cells were stained using the Annexin V-FITC Apoptosis Detection Kit (40302ES60, Yeason, Shanghai, China) and Cell Cycle Detection Kit (KGA512, KeyGEN, Nanjing, China). Lastly, the cell samples were determined by flow cytometry (Beckman, Brea, CA, USA). Data were analyzed by NovoExpress (Agilent, Beijing, China).

### 4.5. Measurement of Mitochondrial Membrane Potential (MMP)

SH-SY5Y and SK-N-AS cells were seeded in 6-well plates and treated with different concentrations of luteoloside (0, 12.5, 25, and 50 μM) for 24 h. After washing the cells three times with PBS, they were stained with JC-1 staining working solution (M8650, Solarbio, Beijing, China) for 20 min at 37 °C in the dark and then detected through flow cytometry (Beckman, Brea, CA, USA). NovoExpress was used to analyze data.

### 4.6. Hoechst 33258 Staining Assay

SH-SY5Y and SK-N-AS cells were cultured in 12-well plates and treated with luteoloside (0, 12.5, 25, and 50 μM) for 24 h. After washing the cells three times with PBS, the cells were fixed with 4% paraformaldehyde solution for 30 min. Next, they were stained with 1 μg/mL Hoechst 33258 (C0021, Solarbio, Beijing, China) for 15 min in the dark, and morphological changes of the nuclei were observed with a fluorescence microscope (Leica, Frankfurt, Germany). Nuclear condensation and fragmentation of cells were considered to indicate apoptotic cells.

### 4.7. qRT-PCR Analysis

RNA extraction reagent was used to extract RNA from SH-SY5Y and SK-N-AS cells. RNA purity and concentration were measured with a UV spectrophotometer according to the instructions. Reverse transcription of RNA to cDNA was performed by a gDNA digester plus (11141ES60, YEASEN, Shanghai, China). Finally, 1 μL of cDNA template, 0.5 μL of primers, and 5 μL of TB Green (RR820A, TaKaRa, Kyoto, Japan) were used for PCR amplification. Each sample was detected using a real-time PCR system (Thermo Fisher Scientific, Billerica, MA, USA). Tubulin served as endogenous control. The primer sequences are listed in Supplementary Table S1.

### 4.8. Western Blot Assay

Radio immunoprecipitation assay buffer (R0010, Solarbio, Beijing, China) was used to lyse the cells for 20 min to extract total protein. The protein concentrations were detected using the Bio-Rad Protein Assay Kit (PC0020, Servicebio, Wuhan, China). Then the proteins were isolated with 12% SDS-PAGE and retransferred onto PVDF membranes (Meilunbio, Dalian, China). The membranes were blocked with 5% skimmed milk for 2 h and incubated with primary antibodies, including cyclin D1 (26939-1-AP), cyclin E (11554-1-AP), C-myc (10828-1-AP), CDK4 (11026-1-AP), p38 (14064-1-AP), p-P38 (Thr180/Tyr182)-(28796-1-AP), ERK (16443-1-AP), p-ERK (Thr202/Tyr204)-(28733-1-AP), AKT (10176-2-AP), p-AKT (Ser473)-(66444-1-Ig), Bcl-2 (60178-1-lg), and tubulin (11224-1-AP), which were purchased from Proteintech Company (Wuhan, China). JNK (9252) and p-JNK (Thr183/Tyr185)-(9255) were obtained from Cell Signaling Technology (Danvers, MA, USA). Cleaved-caspase3 (A11021) was obtained from ABclonal (Wuhan, China). After washing three times with tris-buffered saline, the secondary antibodies were incubated for 2 h. Band intensity was measured using a chemiluminescence Ultra High Sensitivity ECL Kit (AR1190, Boster, Wuhan, China) and analyzed by the ImageJ software (ImageJ 6.0, Bethesda, MD, USA).

### 4.9. Subcutaneous Tumorigenesis Model

Female BALB/c nude mice (4–6 weeks old, body weight of 16–20 g) were received from the Experimental Animal Center of Guizhou Medical University. SH-SY5Y cells (2 × 10^6^ cells/200 µL) were injected subcutaneously into the right upper flank of the mice. After 1 week, when tumors reached 40–60 mm^3^, the mice were randomly subdivided into the luteoloside and DMSO treatment groups (*n* = 5 per group) and injected with DMSO or luteoloside (25 mg/kg/d), respectively, every 3 days. Meanwhile, 3-day measurements of tumor volume and body weight were taken using vernier calipers. Tumor volume was determined according to the formula: (mm^3^) = (Long × Width^2^)/2.21 days. Later, the mice were killed, and kidney, liver, heart, and tumor tissues were taken from the mouse bodies for further study.

### 4.10. Immunohistochemical Staining (IHC)

To dewax and rehydrate paraffin-embedded tumor tissues from mice, different grades of xylene and ethanol solutions were used. After utilizing sodium citrate to retrieve antigens and blocking endogenous peroxidase activity using H_2_O_2_, the tumor samples were incubated with PCNA (GB11010, 1:400, Servicebio, Wuhan, China) and Ki67 (27309-1-AP, 1:300, Proteintech, Wuhan, China) overnight at 4 °C. According to DAB and hematoxylin staining, the secondary antibody was incubated with the uncombined antibody after washing with PBS. Lastly, observations were made by a microscope (Olympus, Kyoto, Japan).

### 4.11. Hematoxylin–Eosin (HE) Staining Assay

After deparaffinization and rehydration, 1% acid ethanol was applied to 5 m longitudinal sections of the kidney, liver, and heart for 5 min, followed by 5 dips in hematoxylin solution (G1120, Solarbio, Beijing, China) and rinsing in distilled water. After staining with eosin solution for 3 min, the sections were dehydrated with graded alcohol and cleared with xylene. HE staining intensity was determined by a microscope (Olympus, Kyoto, Japan).

### 4.12. Network Pharmacology Analysis

The 2D structure (ID: 126832) of luteoloside was downloaded from the PubChem compound database. To predict all potential luteoloside targets, we imported the 2D structure of luteoloside into SwissTargetPrediction (http://swisstargetprediction.ch/), all luteoloside targets are listed in Supplementary Table S2. The potential luteoloside targets were introduced into Cytoscape (https://cytoscape.org/) to construct the compound target diagram. “Betweenness centrality” indicates the target’s importance in transferring information. We introduced the potential target into DAVID (https://david.ncifcrf.gov/) to obtain KEGG enrichment analysis. A bubble map of enrichment analysis was produced using the R programming language.

### 4.13. Molecular Modelling

The crystal structure of the p38 MAPK protein was obtained from the PDB database. The 2D structure (ID: 126832) of luteoloside was downloaded from the PubChem Compound database and transformed to a 3D structure using the Chem3D software (Chem3D 17.0, Waltham, MA, USA). The crystal structure of the p38 MAPK protein and the 3D structure of luteoloside were input the AutoDock software (AutoDock 4.2.6, Beijing, China). Protein activity pockets were identified by automatic pattern recognition after hydrogenation, dehydration, calculation, and atom type addition. According to the binding score, the binding degree of molecule to protein was judged. The results were visualized by the PyMOL software (PyMOL 2.5, Schrödinger, New York, NY, USA).

### 4.14. Cellular Thermal Shift Assay (CETSA)

SH-SY5Y and SK-N-AS cells were cultured in 10 cm culture dishes for 24 h and then centrifuged at 12,000 rpm for 2 min at 37 °C. Then cells were resuspended with 1 mL of PBS containing 20 mM Tris-HCl, 100 mM NaCl, 5 mM EDTA, 2 mM phenylmethylsulfonyl fluoride (PMSF), 10 ng/mL leupeptin, and 10 μg/mL aprotinin. Three freeze–thaw cycles were performed on the cells. Cells were exposed to liquid nitrogen for 3 min each cycle, then heated at 25 °C for 3 min. After centrifuging at 12,000 rpm at 4 °C for half hour, the supernatants from each group were collected. For the luteoloside-treated groups, luteoloside was treated with a final concentration of 50 μM luteoloside. For the control groups, the same quantity of DMSO was added as before. All groups were heated at 25 °C for an hour. Following this, pairs of control and treatment groups were heated for 3 min at 46, 49, 52, 55, 58, and 61 °C. Finally, the samples were analyzed by Western blot.

### 4.15. Statistical Analysis

The GraphPad Prism software (GraphPad Prism 6.01, San Diego, CA, USA) was used for processing. An unpaired t-test was used to measure the differences between the two groups with a cut-off of *p* < 0.05; a one-way analysis of variance combined with a least significant difference t-test was performed to compare differences between multiple groups with a cut-off of *p* < 0.05.

## Data Availability

Not applicable.

## Supplementary Materials

The following supporting information can be downloaded at: https://www.mdpi.com/article/10.3390/molecules28041748/s1, Table S1: Primer sequences for qRT-PCR Detection; Table S2: All the potential targets of luteoloside.

## Sample Availability

Samples of the compounds are available from the authors.

## Abbreviations

Lu	luteoloside
SB	SB203580
IHC	immunohistochemical staining
MAPK	mitogen-activated protein kinase
HE	hematoxylin–eosin
MMP	mitochondrial membrane potential.
