# Supplementary material for: Luteoloside Induces G0/G1 Phase Arrest of Neuroblastoma Cells by Targeting p38 MAPK
Source: Molecules. 2023 Feb 12;28(4):1748. doi: 10.3390/molecules28041748 (PMC9966487; doi:10.3390/molecules28041748)
Supplement: Supplementary file 1 [file molecules-28-01748-s001.zip › molecules-2178744-SI.pdf]

Supplementary Table S1: Primer Sequences for qRT-PCR Detection

Gene Name	Sequence (5' - 3')	
CyclinD1	Forward Primer	TGGTTGACAAAAGGACGC
	Reverse Primer	TGTTTTACCCTTGTCTCGTTC
C-myc	Forward Primer	AAAGCCACAGCATACATCC
	Reverse Primer	TGTGTTTCAACTGTTCTCGTC
Tubulin	Forward Primer	TGGAGTTCTCTATTTACCCGG
	Reverse Primer	CCATGAAGGCACAATCAGAG

Supplementary Table S2: All The Potential Targets of Luteoloside

ID	Gene symbol	Protein
P05177	CYP1A2	Cytochrome P450 1A2
P30543	Adora2a	Adenosine receptor A2a
P31639	SLC5A2	Sodium/glucose cotransporter 2
P25099	Adora1	Adenosine receptor A1
P80457	XDH	Xanthine dehydrogenase/oxidase
O43570	CA12	Carbonic anhydrase 12
Q9ULX7	CA14	Carbonic anhydrase 14
Q16790	CA9	Carbonic anhydrase 9
P14174	MIF	Macrophage migration inhibitory factor
O42713	PPO2	Polyphenol oxidase 2
P16050	ALOX15	Arachidonate 15-lipoxygenase
P13866	SLC5A1	Sodium/glucose cotransporter 1
P11387	TOP1	DNA topoisomerase 1
Q9UNQ0	ABCG2	ATP-binding cassette sub-family G member 2
P43166	CA7	Carbonic anhydrase 7
P14061	HSD17B1	Estradiol 17-beta-dehydrogenase 1
P12527	Alox5	Arachidonate 5-lipoxygenase
P07943	Akr1b1	Aldose reductase
Q8N1Q1	CA13	Carbonic anhydrase 13
O14746	TERT	Telomerase reverse transcriptase
P11309	PIM1	Serine/threonine-protein kinase pim-1
P26358	DNMT1	DNA (cytosine-5)-methyltransferase 1
P23280	CA6	Carbonic anhydrase 6
Q16548	BCL2A1	Bcl-2-related protein A1
P23219	PTGS1	Prostaglandin G/H synthase 1
P51812	RPS6KA3	Ribosomal protein S6 kinase alpha-3
Q92731	ESR2	Estrogen receptor beta

P51452	DUSP3	Dual specificity protein phosphatase 3
P35218	CA5A	Carbonic anhydrase 5A, mitochondrial
P07900	HSP90AA1	Heat shock protein HSP 90-alpha
P18031	PTPN1	Tyrosine-protein phosphatase non-receptor type 1
Q9Y2D0	CA5B	Carbonic anhydrase 5B, mitochondrial
Q9NR96	TLR9	Toll-like receptor 9
P28566	HTR1E	5-hydroxytryptamine receptor 1E
P39900	MMP12	Macrophage metalloelastase
P49759	CLK1	Dual specificity protein kinase CLK1
P05067	APP	Amyloid beta A4 protein
Q9GZU7	CTDSP1	Carboxy-terminal domain RNA polymerase II polypeptide A small phosphatase 1
P02550	TUBA1A	Tubulin alpha-1A chain
O60755	GALR3	Galanin receptor type 3
P15917	lef	Lethal factor
Q07820	MCL1	Induced myeloid leukemia cell differentiation protein Mcl-1
Q9HC97	GPR35	G-protein coupled receptor 35
P21397	MAOA	Amine oxidase [flavin-containing] A
P08183	ABCB1	Multidrug resistance protein 1
P63000	RAC1	Ras-related C3 botulinum toxin substrate 1
Q13627	DYRK1A	Dual specificity tyrosine-phosphorylation-regulated kinase 1A
P24468	NR2F2	COUP transcription factor 2
P35354	PTGS2	Prostaglandin G/H synthase 2
P11511	CYP19A1	Aromatase
Q6B856	TUBB2B	Tubulin beta-2B chain
P29477	Nos2	Nitric oxide synthase, inducible
P30305	CDC25B	M-phase inducer phosphatase 2
P00915	CA1	Carbonic anhydrase 1
P22748	CA4	Carbonic anhydrase 4

Q9Y2R2	PTPN22	Tyrosine-protein phosphatase non-receptor type 22
P36888	FLT3	Receptor-type tyrosine-protein kinase FLT3
P00517	PRKACA	cAMP-dependent protein kinase catalytic subunit alpha
Q04206	RELA	Transcription factor p65
O95136	S1PR2	Sphingosine 1-phosphate receptor 2
P18825	ADRA2C	Alpha-2C adrenergic receptor
P31749	AKT1	RAC-alpha serine/threonine-protein kinase
P34998	CRHR1	Corticotropin-releasing factor receptor 1
P31213	SRD5A2	3-oxo-5-alpha-steroid 4-dehydrogenase 2
P34995	PTGER1	Prostaglandin E2 receptor EP1 subtype
Q9BY41	HDAC8	Histone deacetylase 8
P08575	PTPRC	Receptor-type tyrosine-protein phosphatase C
P08473	MME	Neprilysin
P20272	Cnr1	Cannabinoid receptor 1
Q14524	SCN5A	Sodium channel protein type 5 subunit alpha
P18130	ADRA1A	Alpha-1A adrenergic receptor
O43614	HCRTR2	Orexin receptor type 2
Q05397	PTK2	Focal adhesion kinase 1
O35433	Trpv1	Transient receptor potential cation channel subfamily V member 1
P00760	PRSS1	Cationic trypsin
P51677	CCR3	C-C chemokine receptor type 3
P07858	CTSB	Cathepsin B
Q9Y5Y4	PTGDR2	Prostaglandin D2 receptor 2
Q9H244	P2RY12	P2Y purinoceptor 12
P30291	WEE1	Wee1-like protein kinase
P29089	Agtr1b	Type-1B angiotensin II receptor
P33765	ADORA3	
P33765	TMIGD3	
P25104	AGTR1	Type-1 angiotensin II receptor

P25105	PTAFR	Platelet-activating factor receptor
P14600	Tacr1	Substance-P receptor
P25100	ADRA1D	Alpha-1D adrenergic receptor
P05622	Pdgfrb	Platelet-derived growth factor receptor beta
Q14416	GRM2	Metabotropic glutamate receptor 2
P08842	STS	Steryl-sulfatase
P10827	THRA	Thyroid hormone receptor alpha
P10826	RARB	Retinoic acid receptor beta
Q13085	ACACA	Acetyl-CoA carboxylase 1
P10828	THRB	Thyroid hormone receptor beta
P10275	AR	Androgen receptor
P10276	RARA	Retinoic acid receptor alpha
P56658	ADA	Adenosine deaminase
P19320	VCAM1	Vascular cell adhesion protein 1
Q96EB6	SIRT1	NAD-dependent protein deacetylase sirtuin-1
Q07912	TNK2	Activated CDC42 kinase 1
P19327	Htr1a	5-hydroxytryptamine receptor 1A
O00767	SCD	Acyl-CoA desaturase
O43193	MLNR	Motilin receptor
O00763	ACACB	Acetyl-CoA carboxylase 2
O00408	PDE2A	cGMP-dependent 3',5'-cyclic phosphodiesterase
Q9UM73	ALK	ALK tyrosine kinase receptor
Q01727	Mc1r	Melanocyte-stimulating hormone receptor
P18084	ITGB5	Integrin beta-5
P08253	MMP2	72 kDa type IV collagenase
P80365	HSD11B2	Corticosteroid 11-beta-dehydrogenase isozyme 2
P25929	NPY1R	Neuropeptide Y receptor type 1
P18089	ADRA2B	Alpha-2B adrenergic receptor
P35368	ADRA1B	Alpha-1B adrenergic receptor

P35367	HRH1	Histamine H1 receptor
Q15722	LTB4R	Leukotriene B4 receptor 1
P21451	Ednrb	Endothelin B receptor
P21452	TACR2	Substance-K receptor
P21453	S1PR1	Sphingosine 1-phosphate receptor 1
Q494W8	CHRFAM7A	CHRNA7-FAM7A fusion protein
Q02156	PRKCE	Protein kinase C epsilon type
Q8TDS4	HCAR2	Hydroxycarboxylic acid receptor 2
P36544	CHRNA7	Neuronal acetylcholine receptor subunit alpha-7
Q04631	Fnta	Protein farnesyltransferase/geranylgeranyltransferase type-1 subunit alpha
P12268	IMPDH2	Inosine-5'-monophosphate dehydrogenase 2
P50052	AGTR2	Type-2 angiotensin II receptor
P19099	CYP11B2	Cytochrome P450 11B2, mitochondrial
P49286	MTNR1B	Melatonin receptor type 1B
P13497	BMP1	Bone morphogenetic protein 1
P04035	HMGCR	3-hydroxy-3-methylglutaryl-coenzyme A reductase
P46098	HTR3A	5-hydroxytryptamine receptor 3A
P06401	PGR	Progesterone receptor
O14672	ADAM10	Disintegrin and metalloproteinase domain-containing protein 10
Q9P1W9	PIM2	Serine/threonine-protein kinase pim-2
P23795	ACHE	Acetylcholinesterase
Q92847	GHSR	Growth hormone secretagogue receptor type 1
P50579	METAP2	Methionine aminopeptidase 2
P37268	FDFT1	Squalene synthase
P09960	LTA4H	Leukotriene A-4 hydrolase
Q9R0C9	Sigmar1	Sigma non-opioid intracellular receptor 1
P25774	CTSS	Cathepsin S
Q5S007	LRRK2	Leucine-rich repeat serine/threonine-protein kinase 2

P30729	Drd4	D(4) dopamine receptor
P34972	CNR2	Cannabinoid receptor 2
P37288	AVPR1A	Vasopressin V1a receptor
Q04759	PRKCQ	Protein kinase C theta type
P23458	JAK1	Tyrosine-protein kinase JAK1
P25025	CXCR2	C-X-C chemokine receptor type 2
P25024	CXCR1	C-X-C chemokine receptor type 1
P41968	MC3R	Melanocortin receptor 3
P25021	HRH2	Histamine H2 receptor
P12822	ACE	Angiotensin-converting enzyme
P04049	RAF1	RAF proto-oncogene serine/threonine-protein kinase
P00750	PLAT	Tissue-type plasminogen activator
P43119	PTGIR	Prostacyclin receptor
Q02750	MAP2K1	Dual specificity mitogen-activated protein kinase kinase 1
P32240	Ptger4	Prostaglandin E2 receptor EP4 subtype
P52333	JAK3	Tyrosine-protein kinase JAK3
P55245	EGFR	Epidermal growth factor receptor
P04626	ERBB2	Receptor tyrosine-protein kinase erbB-2
P11802	CDK4	Cyclin-dependent kinase 4
P04629	NTRK1	High affinity nerve growth factor receptor
P54760	EPHB4	Ephrin type-B receptor 4
P05106	ITGB3	Integrin beta-3
Q13946	PDE7A	High affinity cAMP-specific 3',5'-cyclic phosphodiesterase 7A
P17706	PTPN2	Tyrosine-protein phosphatase non-receptor type 2
P51679	CCR4	C-C chemokine receptor type 4
P56817	BACE1	Beta-secretase 1
Q12809	KCNH2	Potassium voltage-gated channel subfamily H member 2
Q00535	CDK5	Cyclin-dependent-like kinase 5
P13631	RARG	Retinoic acid receptor gamma

O43497	CACNA1G	Voltage-dependent T-type calcium channel subunit alpha-1G
P11362	FGFR1	Fibroblast growth factor receptor 1
P41144	OPRK1	Kappa-type opioid receptor
P41146	OPRL1	Nociceptin receptor
Q15761	NPY5R	Neuropeptide Y receptor type 5
P41143	OPRD1	Delta-type opioid receptor
Q8IXJ6	SIRT2	NAD-dependent protein deacetylase sirtuin-2
P33032	MC5R	Melanocortin receptor 5
P21918	DRD5	D(1B) dopamine receptor
P78396	CCNA1	Cyclin-A1
P42866	Oprm1	Mu-type opioid receptor
P43088	PTGFR	Prostaglandin F2-alpha receptor
P48147	PREP	Prolyl endopeptidase
P48145	NPBWR1	Neuropeptides B/W receptor type 1
P14740	Dpp4	Dipeptidyl peptidase 4
P21980	TGM2	Protein-glutamine gamma-glutamyltransferase 2
P35610	SOAT1	Sterol O-acyltransferase 1
P42345	MTOR	Serine/threonine-protein kinase mTOR
Q9Y243	AKT3	RAC-gamma serine/threonine-protein kinase
Q00788	Avpr2	Vasopressin V2 receptor
P10980	Chrm2	Muscarinic acetylcholine receptor M2
P08069	IGF1R	Insulin-like growth factor 1 receptor
P23385	Grm1	Metabotropic glutamate receptor 1
O60725	ICMT	Protein-S-isoprenylcysteine O-methyltransferase
P22460	KCNA5	Potassium voltage-gated channel subfamily A member 5
P05362	ICAM1	Intercellular adhesion molecule 1
P62993	GRB2	Growth factor receptor-bound protein 2
P05364	ampC	Beta-lactamase

P48736	PIK3CG	Phosphatidylinositol 4,5-bisphosphate 3-kinase catalytic subunit gamma isoform
Q02127	DHODH	Dihydroorotate dehydrogenase (quinone), mitochondrial
P00374	DHFR	Dihydrofolate reductase
P16234	PDGFRA	Platelet-derived growth factor receptor alpha
P28702	RXRB	Retinoic acid receptor RXR-beta
P43405	SYK	Tyrosine-protein kinase SYK
P08588	ADRB1	Beta-1 adrenergic receptor
P43403	ZAP70	Tyrosine-protein kinase ZAP-70
Q00960	Grin2b	Glutamate receptor ionotropic, NMDA 2B
P08581	MET	Hepatocyte growth factor receptor
P07711	CTSL	Cathepsin L1
Q03181	PPARD	Peroxisome proliferator-activated receptor delta
P49137	MAPKAPK2	MAP kinase-activated protein kinase 2
P40238	MPL	Thrombopoietin receptor
P34913	EPHX2	Bifunctional epoxide hydrolase 2
Q06187	BTK	Tyrosine-protein kinase BTK
P20288	DRD2	D(2) dopamine receptor
Q13639	HTR4	5-hydroxytryptamine receptor 4
P36897	TGFBR1	TGF-beta receptor type-1
P01375	TNF	Tumor necrosis factor
P27338	MAOB	Amine oxidase [flavin-containing] B
Q99705	MCHR1	Melanin-concentrating hormone receptor 1
Q62053	Ptger2	Prostaglandin E2 receptor EP2 subtype
P08311	CTSG	Cathepsin G
Q9Y5Z0	BACE2	Beta-secretase 2
P25101	EDNRA	Endothelin-1 receptor
Q13258	PTGDR	Prostaglandin D2 receptor
P29371	TACR3	Neuromedin-K receptor

P25116	F2R	Proteinase-activated receptor 1
P30939	HTR1F	5-hydroxytryptamine receptor 1F
P08912	CHRM5	Muscarinic acetylcholine receptor M5
P08913	ADRA2A	Alpha-2A adrenergic receptor
O43613	HCRTR1	Orexin receptor type 1
P49810	PSEN2	Presenilin-2
P29275	ADORA2B	Adenosine receptor A2b
P41180	CASR	Extracellular calcium-sensing receptor
P31391	SSTR4	Somatostatin receptor type 4
Q14790	CASP8	Caspase-8
Q15077	P2RY6	P2Y purinoceptor 6
Q13464	ROCK1	Rho-associated protein kinase 1
Q02763	TEK	Angiopoietin-1 receptor
Q15078	CDK5R1	Cyclin-dependent kinase 5 activator 1
P24941	CDK2	Cyclin-dependent kinase 2
P49355	FNTB	Protein farnesyltransferase subunit beta
P05093	CYP17A1	Steroid 17-alpha-hydroxylase/17,20 lyase
P07478	PRSS2	Trypsin-2
P29476	Nos1	Nitric oxide synthase, brain
P29474	NOS3	Nitric oxide synthase, endothelial
P28221	HTR1D	5-hydroxytryptamine receptor 1D
P28222	HTR1B	5-hydroxytryptamine receptor 1B
P28223	HTR2A	5-hydroxytryptamine receptor 2A
P08631	HCK	Tyrosine-protein kinase HCK
Q15759	MAPK11	Mitogen-activated protein kinase 11
P29597	TYK2	Non-receptor tyrosine-protein kinase TYK2
P19634	SLC9A1	Sodium/hydrogen exchanger 1
Q08881	ITK	Tyrosine-protein kinase ITK/TSK
P19793	RXRA	Retinoic acid receptor RXR-alpha

P07333	CSF1R	Macrophage colony-stimulating factor 1 receptor
P07339	CTSD	Cathepsin D
P21728	DRD1	D(1A) dopamine receptor
P08709	F7	Coagulation factor VII
P14555	PLA2G2A	Phospholipase A2, membrane associated
P47900	P2RY1	P2Y purinoceptor 1
P35968	KDR	Vascular endothelial growth factor receptor 2
P35557	GCK	Glucokinase
P08684	CYP3A4	Cytochrome P450 3A4
Q9HC16	APOBEC3G	DNA dC->dU-editing enzyme APOBEC-3G
P52732	KIF11	Kinesin-like protein KIF11
O60674	JAK2	Tyrosine-protein kinase JAK2
P30559	OXTR	Oxytocin receptor
P30411	BDKRB2	B2 bradykinin receptor
Q99500	S1PR3	Sphingosine 1-phosphate receptor 3
P30553	Cckbr	Gastrin/cholecystokinin type B receptor
P30551	Cckar	Cholecystokinin receptor type A
P30557	Ptger3	Prostaglandin E2 receptor EP3 subtype
P42892	ECE1	Endothelin-converting enzyme 1
P98170	XIAP	E3 ubiquitin-protein ligase XIAP
P56450	Mc4r	Melanocortin receptor 4
P20444	Prkca	Protein kinase C alpha type
P05556	ITGB1	Integrin beta-1
P06737	PYGL	Glycogen phosphorylase, liver form
P53350	PLK1	Serine/threonine-protein kinase PLK1
P45984	MAPK9	Mitogen-activated protein kinase 9
P53609	PGGT1B	Geranylgeranyl transferase type-1 subunit beta
Q13133	NR1H3	Oxysterols receptor LXR-alpha
P45983	MAPK8	Mitogen-activated protein kinase 8

P04054	PLA2G1B	Phospholipase A2
Q04609	FOLH1	Glutamate carboxypeptidase 2
P08235	NR3C2	Mineralocorticoid receptor
P23443	RPS6KB1	Ribosomal protein S6 kinase beta-1
P00747	PLG	Plasminogen
P00742	F10	Coagulation factor X
Q5RAG0	HDAC1	Histone deacetylase 1
P00749	PLAU	Urokinase-type plasminogen activator
P41279	MAP3K8	Mitogen-activated protein kinase kinase kinase 8
Q9H228	S1PR5	Sphingosine 1-phosphate receptor 5
P20701	ITGAL	Integrin alpha-L
P47901	AVPR1B	Vasopressin V1b receptor
Q7TMR0	Prcp	Lysosomal Pro-X carboxypeptidase
P22001	KCNA3	Potassium voltage-gated channel subfamily A member 3
Q00G26	PLIN5	Perilipin-5
P49682	CXCR3	C-X-C chemokine receptor type 3
P49841	GSK3B	Glycogen synthase kinase-3 beta
P49840	GSK3A	Glycogen synthase kinase-3 alpha
P97612	Faah	Fatty-acid amide hydrolase 1
Q9QYN8	Hrh3	Histamine H3 receptor
P35869	AHR	Aryl hydrocarbon receptor
P23141	CES1	Liver carboxylesterase 1
O14939	PLD2	Phospholipase D2
P48039	MTNR1A	Melatonin receptor type 1A
P31941	APOBEC3A	DNA dC->dU-editing enzyme APOBEC-3A
Q8TDV5	GPR119	Glucose-dependent insulinotropic receptor
P23978	Slc6a1	Sodium- and chloride-dependent GABA transporter 1
O15379	HDAC3	Histone deacetylase 3
Q05469	LIPE	Hormone-sensitive lipase

Q9Y271	CYSLTR1	Cysteinyl leukotriene receptor 1
P35916	FLT4	Vascular endothelial growth factor receptor 3
Q86V86	PIM3	Serine/threonine-protein kinase pim-3
Q16602	CALCRL	Calcitonin gene-related peptide type 1 receptor
P08173	CHRM4	Muscarinic acetylcholine receptor M4
P53779	MAPK10	Mitogen-activated protein kinase 10
P32247	BRS3	Bombesin receptor subtype-3
P32246	CCR1	C-C chemokine receptor type 1
P00491	PNP	Purine nucleoside phosphorylase
P35346	SSTR5	Somatostatin receptor type 5
P00797	REN	Renin
O14684	PTGES	Prostaglandin E synthase
P78536	ADAM17	Disintegrin and metalloproteinase domain-containing protein 17
O15530	PDPK1	3-phosphoinositide-dependent protein kinase 1
P13516	Scd1	Acyl-CoA desaturase 1
Q00975	CACNA1B	Voltage-dependent N-type calcium channel subunit alpha-1B
P32745	SSTR3	Somatostatin receptor type 3
P50172	Hsd11b1	Corticosteroid 11-beta-dehydrogenase isozyme 1
P33261	CYP2C19	Cytochrome P450 2C19
P08254	MMP3	Stromelysin-1
Q64663	P2rx7	P2X purinoceptor 7
P35462	DRD3	D(3) dopamine receptor
P47898	HTR5A	5-hydroxytryptamine receptor 5A
Q9Y5X4	NR2E3	Photoreceptor-specific nuclear receptor
P04150	NR3C1	Glucocorticoid receptor
P34969	HTR7	5-hydroxytryptamine receptor 7
Q01959	SLC6A3	Sodium-dependent dopamine transporter
P20292	ALOX5AP	Arachidonate 5-lipoxygenase-activating protein
P08482	Chrm1	Muscarinic acetylcholine receptor M1

P08483	Chrm3	Muscarinic acetylcholine receptor M3
P47871	GCGR	Glucagon receptor
O96020	CCNE2	G1/S-specific cyclin-E2
P23975	SLC6A2	Sodium-dependent noradrenaline transporter
O96017	CHEK2	Serine/threonine-protein kinase Chk2
P41231	P2RY2	P2Y purinoceptor 2
O96013	PAK4	Serine/threonine-protein kinase PAK 4
P41235	HNF4A	Hepatocyte nuclear factor 4-alpha
P19156	ATP4A	Potassium-transporting ATPase alpha chain 1
P56524	HDAC4	Histone deacetylase 4
P18405	SRD5A1	3-oxo-5-alpha-steroid 4-dehydrogenase 1
Q96RI1	NR1H4	Bile acid receptor
Q6V1X1	DPP8	Dipeptidyl peptidase 8
P55211	CASP9	Caspase-9
P55210	CASP7	Caspase-7
P55212	CASP6	Caspase-6
P51681	CCR5	C-C chemokine receptor type 5
P51685	CCR8	C-C chemokine receptor type 8
P08909	Htr2c	5-hydroxytryptamine receptor 2C
Q07343	PDE4B	cAMP-specific 3',5'-cyclic phosphodiesterase 4B
P31645	SLC6A4	Sodium-dependent serotonin transporter
Q96RJ0	TAAR1	Trace amine-associated receptor 1
P31424	Grm5	Metabotropic glutamate receptor 5
P31388	Htr6	5-hydroxytryptamine receptor 6
P15144	ANPEP	Aminopeptidase N
P05129	PRKCG	Protein kinase C gamma type
P09483	Chrna4	Neuronal acetylcholine receptor subunit alpha-4
P07948	LYN	Tyrosine-protein kinase Lyn
P07949	RET	Proto-oncogene tyrosine-protein kinase receptor Ret

O95180	CACNA1H	Voltage-dependent T-type calcium channel subunit alpha-1H
P16581	SELE	E-selectin
Q969F8	KISS1R	KiSS-1 receptor
O00748	CES2	Cocaine esterase
P14780	MMP9	Matrix metalloproteinase-9
P29466	CASP1	Caspase-1
P61073	CXCR4	C-X-C chemokine receptor type 4
P28482	p38 MAPK	p38 Mitogen-activated protein kinase
Q14432	PDE3A	cGMP-inhibited 3',5'-cyclic phosphodiesterase A
P06493	CDK1	Cyclin-dependent kinase 1
P08238	HSP90AB1	Heat shock protein HSP 90-beta
Q9HAZ1	CLK4	Dual specificity protein kinase CLK4
P13945	ADRB3	Beta-3 adrenergic receptor
P00918	CA2	Carbonic anhydrase 2
O95822	MLYCD	Malonyl-CoA decarboxylase, mitochondrial
P48067	SLC6A9	Sodium- and chloride-dependent glycine transporter 1
Q9QYJ6	Pde10a	cAMP and cAMP-inhibited cGMP 3',5'-cyclic phosphodiesterase 10A
P21731	TBXA2R	Thromboxane A2 receptor
P35398	RORA	Nuclear receptor ROR-alpha
Q8TDU6	GPBAR1	G-protein coupled bile acid receptor 1
P08514	ITGA2B	Integrin alpha-IIb
P10635	CYP2D6	Cytochrome P450 2D6
P49146	NPY2R	Neuropeptide Y receptor type 2
P30530	AXL	Tyrosine-protein kinase receptor UFO
P46663	BDKRB1	B1 bradykinin receptor
P35236	PTPN7	Tyrosine-protein phosphatase non-receptor type 7
P30874	SSTR2	Somatostatin receptor type 2
P15538	CYP11B1	Cytochrome P450 11B1, mitochondrial
P30872	SSTR1	Somatostatin receptor type 1

P14324	FDPS	Farnesyl pyrophosphate synthase
P16257	Tspo	Translocator protein
P06239	LCK	Tyrosine-protein kinase Lck
P50750	CDK9	Cyclin-dependent kinase 9
P68400	CSNK2A1	Casein kinase II subunit alpha
P31751	AKT2	RAC-beta serine/threonine-protein kinase
P24557	TBXAS1	Thromboxane-A synthase
Q9GZT9	EGLN1	Egl nine homolog 1
P13726	F3	Tissue factor
P35439	Grin1	Glutamate receptor ionotropic, NMDA 1
Q8WW43	APH1B	Gamma-secretase subunit APH-1B
Q8T6T2	56k.02	Inosine-5'-monophosphate dehydrogenase
P07607	Tyms	Thymidylate synthase
P42262	GRIA2	Glutamate receptor 2
P03956	MMP1	Interstitial collagenase
P10415	BCL2	Apoptosis regulator Bcl-2
P55263	ADK	Adenosine kinase
O00311	CDC7	Cell division cycle 7-related protein kinase
Q99685	MGLL	Monoglyceride lipase
O60240	PLIN1	Perilipin-1
Q05655	PRKCD	Protein kinase C delta type
P60953	CDC42	Cell division control protein 42 homolog
P13612	ITGA4	Integrin alpha-4
O14842	FFAR1	Free fatty acid receptor 1
Q15661	TPSAB1	Tryptase alpha/beta-1
O43353	RIPK2	Receptor-interacting serine/threonine-protein kinase 2
O14920	IKBKB	Inhibitor of nuclear factor kappa-B kinase subunit beta
O75116	ROCK2	Rho-associated protein kinase 2
Q15858	SCN9A	Sodium channel protein type 9 subunit alpha

Q9NZ42	PSENEN	Gamma-secretase subunit PEN-2
P11086	PNMT	Phenylethanolamine N-methyltransferase
P11712	CYP2C9	Cytochrome P450 2C9
Q86TI2	DPP9	Dipeptidyl peptidase 9
P08246	ELANE	Neutrophil elastase
Q9H3N8	HRH4	Histamine H4 receptor
P17948	FLT1	Vascular endothelial growth factor receptor 1
Q92769	HDAC2	Histone deacetylase 2
P55157	MTTP	Microsomal triglyceride transfer protein large subunit
Q9NWZ3	IRAK4	Interleukin-1 receptor-associated kinase 4
P05121	SERPINE1	Plasminogen activator inhibitor 1
Q96BI3	APH1A	Gamma-secretase subunit APH-1A
Q92542	NCSTN	Nicastrin
P37231	PPARG	Peroxisome proliferator-activated receptor gamma
P42338	PIK3CB	Phosphatidylinositol 4,5-bisphosphate 3-kinase catalytic subunit beta isoform
P42336	PIK3CA	Phosphatidylinositol 4,5-bisphosphate 3-kinase catalytic subunit alpha isoform
P45452	MMP13	Collagenase 3
P22894	MMP8	Neutrophil collagenase
P42330	AKR1C3	Aldo-keto reductase family 1 member C3
P30989	NTSR1	Neurotensin receptor type 1
Q08499	PDE4D	cAMP-specific 3',5'-cyclic phosphodiesterase 4D
P81908	BCHE	Cholinesterase
Q14833	GRM4	Metabotropic glutamate receptor 4
P00734	F2	Prothrombin
P23946	CMA1	Chymase
P43235	CTSK	Cathepsin K
Q99808	SLC29A1	Equilibrative nucleoside transporter 1

Q9UBN7	HDAC6	Histone deacetylase 6
P50613	CDK7	Cyclin-dependent kinase 7
Q9UHL4	DPP7	Dipeptidyl peptidase 2
P48443	RXRG	Retinoic acid receptor RXR-gamma
Q00987	MDM2	E3 ubiquitin-protein ligase Mdm2
P46925	PMII	Plasmepsin-2
P05771	PRKCB	Protein kinase C beta type
Q13093	PLA2G7	Platelet-activating factor acetylhydrolase
P06756	ITGAV	Integrin alpha-V
P20231	TPSB2	Tryptase beta-2
O14965	AURKA	Aurora kinase A
P62943	FKBP1A	Peptidyl-prolyl cis-trans isomerase FKBP1A
P41597	CCR2	C-C chemokine receptor type 2
P16753	UL80	Capsid scaffolding protein
P09874	PARP1	Poly [ADP-ribose] polymerase 1
P07384	CAPN1	Calpain-1 catalytic subunit
O00329	PIK3CD	Phosphatidylinositol 4,5-bisphosphate 3-kinase catalytic subunit delta isoform
P27815	PDE4A	cAMP-specific 3',5'-cyclic phosphodiesterase 4A
Q16539	MAPK14	Mitogen-activated protein kinase 14
Q28156	PDE5A	cGMP-specific 3',5'-cyclic phosphodiesterase
Q06418	TYRO3	Tyrosine-protein kinase receptor TYRO3
P30968	GNRHR	Gonadotropin-releasing hormone receptor
P11597	CETP	Cholesteryl ester transfer protein
Q07817	BCL2L1	Bcl-2-like protein 1
P09237	MMP7	Matrilysin
Q07869	PPARA	Peroxisome proliferator-activated receptor alpha
P00519	ABL1	Tyrosine-protein kinase ABL1
P05186	ALPL	Alkaline phosphatase, tissue-nonspecific isozyme

O95977	S1PR4	Sphingosine 1-phosphate receptor 4
O75907	DGAT1	Diacylglycerol O-acyltransferase 1
Q99835	SMO	Smoothened homolog
P49768	PSEN1	Presenilin-1
P09958	FURIN	Furin
P15056	BRAF	Serine/threonine-protein kinase B-raf
Q9UKP6	UTS2R	Urotensin-2 receptor
P06241	FYN	Tyrosine-protein kinase Fyn
P42574	CASP3	Caspase-3
P37059	HSD17B2	Estradiol 17-beta-dehydrogenase 2
P37058	HSD17B3	Testosterone 17-beta-dehydrogenase 3
